# Not all plant-based diets are associated with benefits on mortality: the Moli-sani Study: Marialaura Bonaccio

**DOI:** 10.1093/eurpub/ckac129.172

**Published:** 2022-10-25

**Authors:** M Bonaccio, A Di Castelnuovo, S Costanzo, E Ruggiero, S Esposito, M Persichillo, C Cerletti, MB Donati, G de Gaetano, L Iacoviello

**Affiliations:** 1 Department of Epidemiology and Prevention, IRCCS NEUROMED, Pozzilli, Italy; 2 Cardiocentro, Clinica Mediterranea, Naples, Italy; 3 Department of Medicine and Surgery, University of Insubria, Varese, Italy

## Abstract

**Background:**

Vegetarians diets are characterized by the absence of some animal foods (e.g. red and processed meats), and a high consumption of plant-based foods. However, plant-based foods can include foods with varying nutritional value and health effects. We examined the association of three different pro-vegetarian (PVG) food patterns defined as general (gPVG), healthful (hPVG) and unhealthful (uPVG), with the risk of all-cause and cardiovascular disease (CVD) mortality in Italians.

**Methods:**

Longitudinal analysis on 22,912 men and women (mean age 55±12 y) from the Moli-sani Study (2005-2010) followed up for 11.2 y (median). Food intake was assessed by a 188-item FFQ. A provegetarian food pattern (FP) was constructed by assigning positive scores to plant foods and reverse scores to animal foods. A healthful and an unhealthful pro-vegetarian FP, which distinguished between healthy (e.g. fruits, vegetables, legumes) and less-healthy plant foods (e.g. fruit juices, potatoes, sugary beverages), were also built up.

**Results:**

In multivariable-adjusted analyses controlled for known risk factors, higher adherence to a gPVG was associated with lower all-cause (HR = 0.83; 95%CI 0.73-0.94) but not CVD mortality (HR = 0.90; 0.72-1.12). Increasing adherence to a hPVG was associated with reduced all-cause mortality risk (HR = 0.82; 0.72-0.95) as well as lower risk of CVD mortality (HR = 0.75; 0.59-0.95). Finally, the uPVG was directly associated with both all-cause (HR = 1.17; 1.03-1.33) and CVD mortality risks (HR = 1.23; 0.99-1.53).

**Conclusions:**

A general pro-vegetarian food pattern was associated with longer survival in Italians. Preferring healthful vegetarian foods provided protection against CVD mortality too. Consistently, a large dietary share of unhealthful vegetarian foods, mostly highly processed, was associated with increased risk mortality. Thus the quality of the plant food consumed is paramount to achieve diet-related benefits on mortality.

**Key messages:**

• A pro-vegetarian food pattern was associated with longer survival but preferring healthful vegetarian foods provided protection against CVD mortality too.

• The quality of the plant food consumed is paramount to achieve diet-related benefits on mortality.

